# Intramedullary cervical spinal cord teratoma

**DOI:** 10.1097/MD.0000000000020107

**Published:** 2020-05-01

**Authors:** Lishuai Wang, Tongxiang Li, Min Gong, Fei Xing, Lang Li, Rui Xiao, Qing Guan

**Affiliations:** a Department of Oncology, The Second People's Hospital of Yibin; b Department of Orthopedics, The First People's Hospital of Yibin, Yibin City; c Department of Orthopedics, Chengdu; d Department of Pediatric surgery, West China Hospital, Sichuan University, Chengdu, Sichuan Province, PR China.

**Keywords:** cervical spine, intramedullary tumor, quadriparesis, teretoma

## Abstract

**Background:**

Intramedullary cervical spinal cord teratomas (ICTs) are extremely rare, and diagnosis and treatment are challenging. We conducted a systematic review of the literature on the diagnosis and treatment of ICT.

**Method:**

The presentation, imaging manifestations, diagnosis, management, surgery findings, prognosis and histology were reviewed following Preferred Reporting Items for Systematic Reviews and Meta Analyses guidelines. English-language studies and case reports published from inception to 2018 were retrieved. Data on presentation, imaging characteristics, diagnosis, management, surgery findings, outcomes, and histopathology were extracted.

**Results:**

Ten articles involving 10 patients were selected. The lesions were located in the upper cervical vertebrae in 4 cases, whereas in the lower cervical vertebrae in the remaining 6 cases. In 5 cases, the lesions were located on the dorsal side of the spinal cord, and in the center of the spinal cord in the remaining 5 cases. Quadriparesis (60%), paraplegia (30%), monoplegia (10%), and neck pain (50%) were the main presentations. The lesion appeared as a intramedullary heterogeneous signal during an MRI scan, and the lesion signal would be partially enhanced after the contrast medium was applied. All patients underwent surgical intervention through a posterior approach. Neurological function improved postoperatively in all patients. Two patients with pathology confirmed to be immature teratomas experienced recurrence.

**Conclusion:**

ICTs are extremely rare entities that are mainly located in the center or dorsal part of the spinal cord which mainly manifest as quadriplegia and neck pain. MRI is a useful modality that provides diagnostic clues. Surgery from a posterior approach is the primary treatment, and the effect of adjuvant therapy remains uncertain. The prognosis is mainly related to the pathological nature of the tumor and not the method of resection.

## 1 Introduction

Teratomas account for about 0.1% of all spinal tumors, and intramedullary spinal cord teratomas, which are less common, are found mainly in the lumbar and thoracic segments.^[1,2,3,4,5]^ Intramedullary cervical spinal cord teratomas (ICTs), first reported by Dereymaeker in 1954, are extremely rare, with only a small number of case reports found in the literature.^[6,7,8]^ ICTs often manifest as myelopathy because of the involvement of the spinal cord, and the diagnosis and treatment of ICTs remain challenging due to the lack of specific symptoms or signs and the unique location of the tumor. A majority of the studies describing ICTs are case reports, and there are no systematic reviews. Therefore, we conducted a systematic review of the ICTs to provide a summary on their incidence and prevalence rates, presentation, imaging characteristics, diagnosis, management, prognosis, and histological characteristics.

## 2 Materials and methods

### 2.1 Literature search

The systematic review was conducted following the PRISMA (Preferred Reporting Items for Systematic Reviews and Meta-Analyses).^[9]^ Potentially relevant publication was retrieved from PubMed, Embase, and the Cochrane library from inception to 2018. The search strategy included combinations of the terms “intramedullary teratoma,” “spine teratoma,” cervical teratoma,” either as keywords or as MeSH terms. The reference lists of retrieved literatures were manually searched for relevant articles, and the abstracts were read for possible full text review and inclusion. Articles were screened and selected independently by 2 reviewers. Disagreements were resolved by discussion, and a third author conducted an independent review if agreement was not reached.

### 2.2 Inclusion and exclusion criteria

The full text of articles written in English, published after 1980, and involving human subjects were included. Prospective clinical trials, retrospective studies, reports of case series, and case reports with data on intramedullary cervical teratoma were eligible for inclusion. Cadaver studies, laboratory or animal studies and reports of teratoid tumor were excluded. Meta-analyses and systematic reviews were excluded.

### 2.3 Data extraction

The names of the first and corresponding authors, type of study, publication date, number of patients, presentation, imaging manifestation, lesion level, surgical procedure, findings during surgery, outcome, histology and duration of follow-up were extracted.

## 3 Results

### 3.1 Study selection

**Figure 1:**
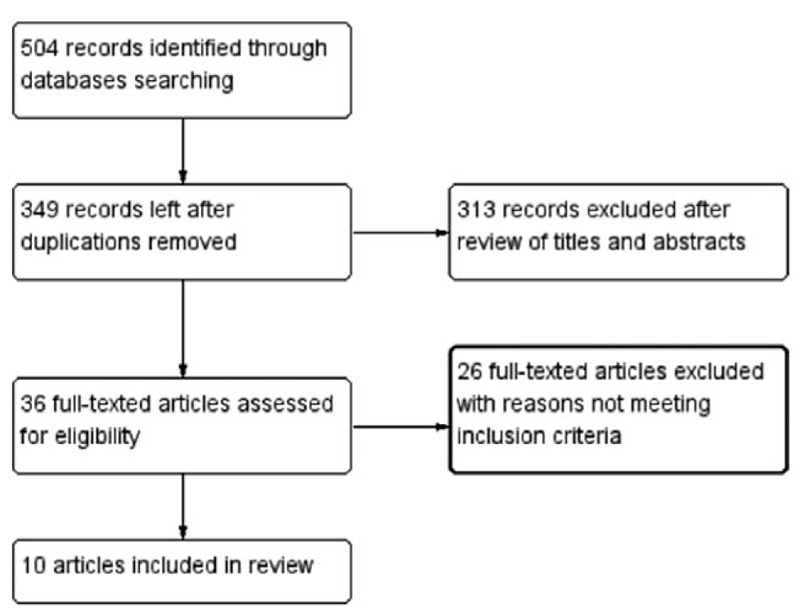
Flow diagram showing selection of studies.

The initial literature search yielded 504 articles. After eliminating duplicates, 349 articles were retained for further screening. Of these, 313 were excluded following a review of the titles and abstracts, and 26 articles were excluded because they did not meet the inclusion criteria. Finally, 10 articles describing ten patients were included in this systematic review.^[7,8,10–17]^Figure 1 shows the selection process.

### 3.2 Study and patient characteristics

All the studies included in the systematic review were case reports, each describing one patient, were published between 1982 and 2015, and included 4 males and 6 females (Table 1). Specifically, 4, 3, and 3 case reports were from Europe, North America, and Asia, respectively. The average patient age was 40.8 (range, 15–65) years. In 4 cases, the lesion was located in the upper cervical vertebrae (C0–C2), whereas the lesion was located in the lower cervical vertebrae (C3-C7) in the remaining 6 cases. In 5 cases, the lesion was located on the dorsal side of the spinal cord, and the lesion in the remaining 5 cases was located in the center of the spinal cord. None of the reported lesions were located on the ventral side of the spinal cord. The average follow-up period was 16.5 months.

**Table 1:**
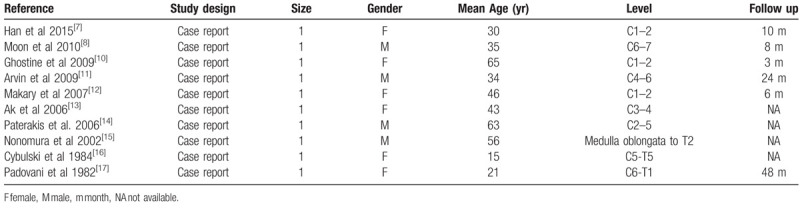
Summary of studies included for review.

### 3.3 Presentation

Quadriparesis, including quadriplegia without pain (30%, n = 3) and quadriplegia combined with neck pain (30%, n = 3), was the most common symptom (60%, n = 6), followed by paraplegia combined with neck pain (20%, n = 2), monoplegia (10%, n = 1), and monoplegia combined with chest pain (10%, n = 1). Neck pain was reported in 50% (n = 5) of the patients. Upon physical examination, 3 patients (30%) were found to have a skin dimple or nodular mass with surrounding hair at the midline of the dorsal cervical region (Table 2).

**Table 2:**
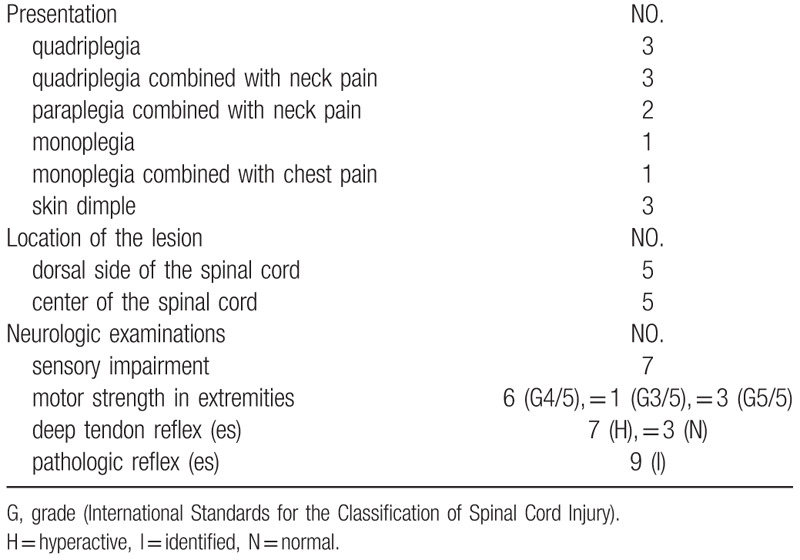
The presentation of patients.

### 3.4 Imaging manifestations

**Figure 2:**
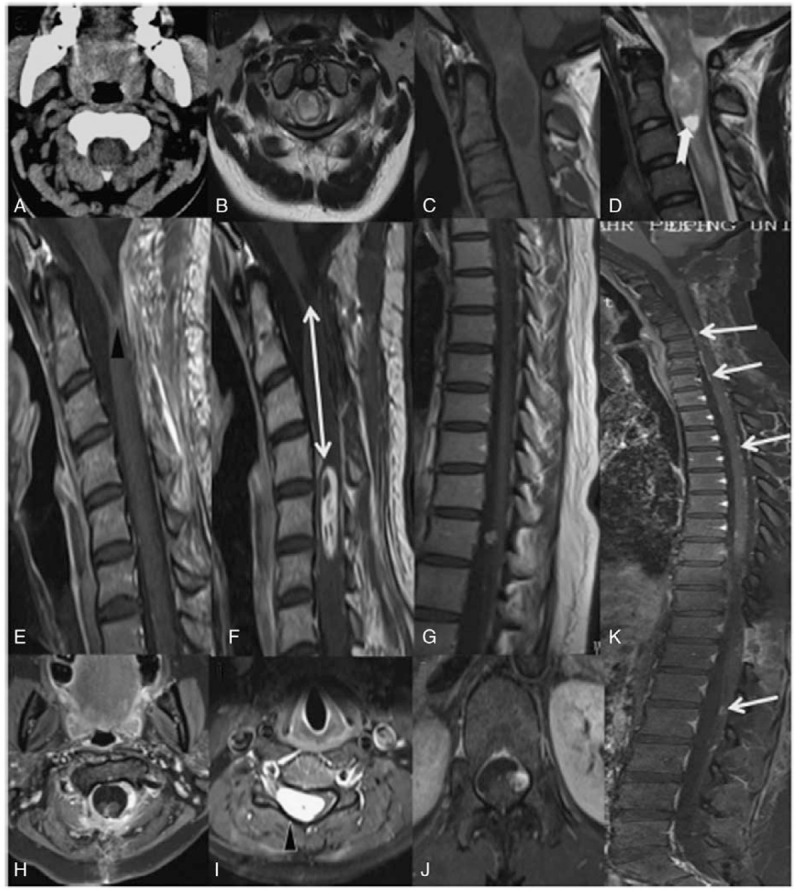
Preoperative plain computed tomography scan shows a hypodense lesion located at C1–C2 levels without calcification (A). Preoperative axial T1-weighted MRI shows the bad-delineated mass of hypointense signal located exclusively in the cervical spinal cord (B). Sagittal MRI (C and D) depicts the fusiform-shaped and eccentrically located intramedullary tumor at the C1–C2 levels, appearing hypointense on T1-weighted and mixed signal intensity on T2-weighted images with adjacent spinal cord edema. The cystic lesion within the spinal cord distal to the mass is quite visible (see white dovetail arrow). (E and H) Immediately postoperative Gd-enhanced T1-weighted sagittal and axial MRI shows gross-total removal of the tumor (black arrowhead). Sagittal (F and G) and axial (I and J) on the 10th month follow-up MRI images with contrast reveal two metastatic extramedullary lesions at C4–C6 and T11–12, respectively, appearing highly enhanced with heterogeneous features. No changes are observed in the previous operation site (see white double arrow). (K) Postoperative T1-weighted sagittal MRI with contrast depicts near-total resection of the cranial and caudal tumors, respectively. Of note, numerous additional disseminated enhancing foci nodules are seen along the leptomeninges and dura, likely representing metastatic drop lesions (long white arrows). (Han Z, Du Y, Qi H, Zheng S, Yin W. Cervical intramedullary immature teratoma with metastatic recurrence in an adult. Spinal Cord Ser Cases. 2015;1:15006.).

Magnetic resonance imaging (MRI), computed tomography (CT), X-ray, and myelography were the most frequently used imaging modalities. In this review, eight patients were evaluated with MRI, whereas the two patients reported in the 1980s underwent myelography and X-ray. CT and X-ray were able to reveal coexisting spinal deformities. Table 3 summarizes the imaging manifestations of the case reports included in this review. Figure 2 is a representative MRI image of Intramedullary teratoma reported by Han et al.^[7]^

**Table 3:**
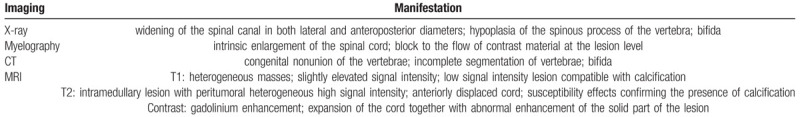
The imaging manifestations of ICTs.

### 3.5 Diagnosis, surgery, and outcomes

None of the lesions were diagnosed as a teratoma before surgery, and all the lesions were confirmed as ICTs by postoperative pathological examination. All the patients underwent surgery after the discovery of the intramedullary lesion, and all the procedures are performed with a posterior approach for laminectomy. Among these 10 cases, subtotal resection, total resection, and partial resection were performed in 5, 4, and 1 case, respectively. Postoperatively, 1 patient suffered cerebrospinal fluid leak at the incision site, which was resolved by oversewing the wound and placing a lumbar drain. The symptoms in all the patients were resolved to different degrees in the early postoperative period. Two patients with immature teratomas had recurrence in the fourth and tenth months after the initial operation, respectively, whereas none of the 8 patients with mature teratomas experienced relapse during a follow-up period of 3 to 48 months (Table 4). One of the relapsed patients underwent reoperation and received chemoradiotherapy.

**Table 4:**
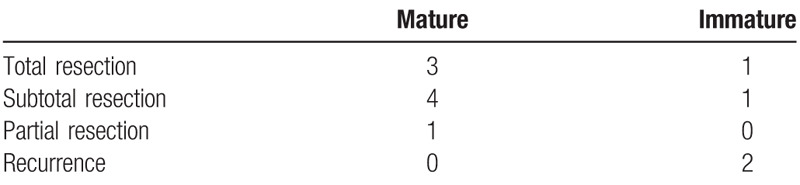
Surgery and outcomes.

### 3.6 Surgical findings

After the laminectomy, an expanded and tense dura mater could be seen at the level of the lesion in most cases. The lesions could be located following durotomy and myelotomy from the posterior midline. In all cases, the tumors appeared to be pink/gray in appearance and rubbery in consistency and were mainly composed of cystic and solid portions. A thick fluid could be observed following the rupture of the cystic portion. In some cases, there was a relative cleavage plane between the lesion and the spinal cord, whereas the lesion was connected to the spinal cord in some parts. Therefore, some lesions could be removed completely, whereas only partial removal could be achieved in other cases to avoid damage to the spinal cord because of total resection. The skin mass or dimples, when present, were preserved, and their tracts were traced during surgery to determine if they entered the dura and were connected to the lesion (Table 5).

**Table 5:**
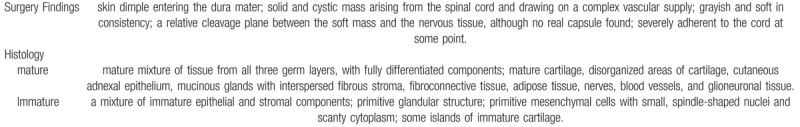
Surgery findings and histology.

### 3.7 Histopathology

**Figure 3:**
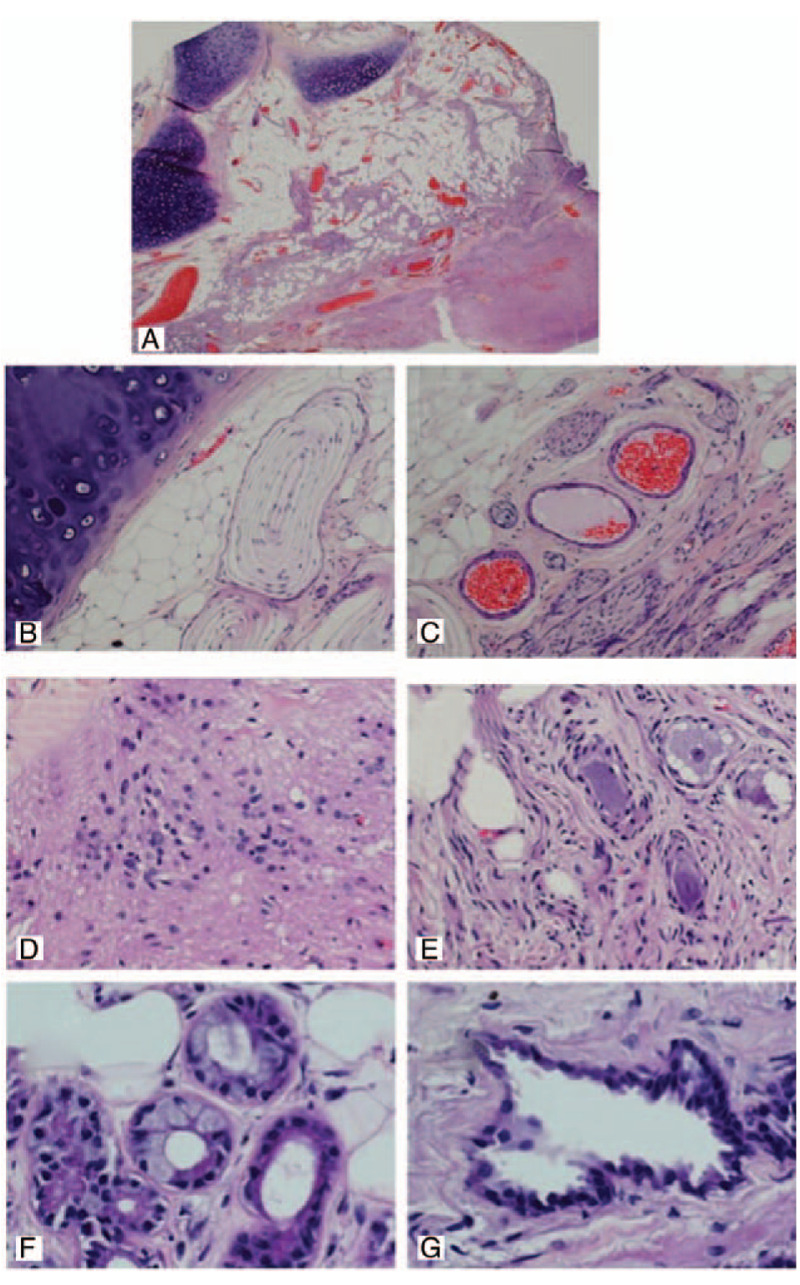
Hematoxylin-eosin stained section showing typical histological appearances. (A) Overview demonstrating cartilage (top left) adipose tissue, vessels, and nerves (center) and neurological tissue (bottom right) (Magnification [mag.]) 12.5×); (B) cartilage, adipose tissue, and pacini corpuscles (mag. 100×);(C) blood vessels and nerves (mag. 100×); (D) neuroglial tissue (mag. 200×); (E) large mature ganglion cells (mag. 200×); (F) serous/mucus glands (mag. 400×); and (G) cyst with cuboidal epithelium (mag. 400×). (Arvin B, Pohl U, David K. Intramedullary cervical teratoma in an adult. The Spine J. 2009;9(5):e14-8.).

Histopathological analysis was performed on all the excised lesions. In mature ICTs, histopathology revealed haphazardly arranged islands of a mature mixture of tissues from all 3 germ layers, including epithelium, fat, cartilage, mucous glands, smooth muscle, myelinated fibers, nerves, and blood vessels. The immature teratomas were composed of immature epithelial and stromal components, including primitive glandular structures and primitive mesenchymal cells (Table 5). There was a representative histopathology picture reported by Arvin et al in 2009 (Fig. 3).^[12]^

## 4 Discussion

Teratomas of the spinal cord are very rare, primarily located in the sacrococcygeal and thoracolumbar regions.^[18–21]^ Depending on the tumor location, they can be classified as extradural and intradural teratomas. The latter can be located extramedullary or intramedullary.^[22,23]^ Cervical ICTs are very rare, as reflected by the exceptionally low number of case reports published to date.

The pathogenesis of intradural spinal teratoma is controversial. The dysembryogenic theory and the misplaced germ cell theory are the two most widely discussed theories.^[24,25,26,27]^ The dysembryogenic theory considers that genetic errors and inductive cellular interactions of the pluripotent cells from the caudal mass of a developing embryo are the origin of teratoma, which is supported by the association of teratomas and dysraphic processes. The misplaced germ cell theory postulates that intradural teratomas arise from the primordial germ cells misplaced in the dorsal midline during their normal migration from the primitive yolk sac to the gonadal ridges. In the current review, 5 patients (50%) with intramedullary teratomas had deformities of the cervical spine, including spinal bifida, incomplete segmentation, congenital nonunion of vertebral body, hypoplasia of the spinous process, and skin dimple on the dorsum of the neck, which provide evidence for the dysembryogenic theory. No spinal deformities were found in the remaining 5 patients. All the tumors included in the current review were located in the central or dorsal part of the cervical spinal cord, supporting the misplaced germ cell theory. A new hypothesis proposes a cause-and-effect relationship between the spinal cord malformations and the migration of the pluripotential cells, which get entrapped in an abnormal environment that leads to teratoma formation.^[12]^

Intramedullary teratomas can be categorized as mature and immature teratomas based on the degree of cell differentiation. A mature teratoma with well differentiated cells shows the characteristics of a benign tumor, while an immature teratoma harboring primitive, immature, and undifferentiated cell characteristics often has the features of malignant tumors.^[28]^ In the current review, there was no recurrence in 8 patients with mature cervical intramedullary teratomas, although some of the tumors were removed with partial or subtotal excision. In contrast, both of the patients with immature teratomas who underwent total and near-total resection suffered relapse.^[7,8]^

The clinical presentations of cervical intramedullary teratomas were variable and related to the location and size of the tumor. The main manifestations were symptoms and signs related to cervical spinal cord injury. Quadriparesis was the primary manifestation and included motor dysfunction and/or sensory changes. The patients presented with varying degrees of strength reduction and deep and shallow sensory disturbances in the extremities. Hyperactive deep tendon reflexes and ankle clonus were present in some patients. In addition, paraplegia and monoplegia were observed in some patients with relatively small tumor volumes. In the current review, the voiding difficulty was reported in one patient only. A more prominent feature of patients with intramedullary teratomas was the relatively high incidence of neck pain, which was reported in 50% of the patients included in the current review. A posterior skin dimple with a hair around in the neck region was an important clue for teratoma.

Evaluation using imaging studies is an important diagnostic tool, which can provide some diagnostic evidence but cannot confirm the diagnosis of teratoma.^[29,30,31]^ X-ray and CT can show changes in the bony structure of the spine due to the presence of tumors, such as widening of the spinal canal, congenital nonunion, incomplete segmentation, and spina bifida. MRI is the most useful imaging method for cervical intramedullary teratomas that can provide information on several characteristics of the tumor such as location and size. On MRI, the lesion is observed as a lobular mass with homogeneous intermediate signal intensity that arises from within the spinal cord and displays an indistinct enhancement with contrast medium. Some of the lesions have cystic cavities that are well displayed in MRI.^[28]^ Intramedullary teratomas of the spinal cord were predominantly located in the lower thoracic and thoracolumbar regions.^[18,21]^ Therefore, it is necessary to perform an MRI scan of the thoracic and lumbar spine when a cervical intramedullary teratoma is found.^[8]^ All the ten literatures did not described the biochemical changes in cerebrospinal fluid and its role in the diagnosis of intramedullary cervical teratoma. We believe that it is necessary to pay attention to research in this area in the future. Teratomas also occur in the intracranial medulla.^[40,41]^ However, we have not found any literature describing the association between intracranial intramedullary teratomas and spinal intramedullary teratomas.

Surgical resection is the primary treatment for ICTs. For intramedullary tumor resection, intraoperative electrophysiological monitoring can reduce the risk of neurological damage.^[33,37,38]^ However, some scholars have pointed out that intraoperative monitoring may limit the scope of tumor resection.^[39]^ The lesions may have solid and cystic components. Therefore, to avoid postoperative aseptic chemical meningitis, surgeons should aim at reducing the spillage of cystic components intraoperatively.^[35,36]^ There may be a firm attachment between the lesion and the spinal structure at certain areas or a relative cleavage plane, although no real capsule was found. Laminectomy or laminoplasty from a posterior approach was applied in all patients reported to date. Following the tract of the skin dimple during surgery may aid in determining the tumor position. Total resection of the tumor without damaging the spinal cord is the surgical goal. However, due to the adhesion of the tumor with the spinal cord during surgery, this may not be fully realized in all patients. To avoid damage to normal spinal cord tissue, subtotal resection is sometimes required.^[13,32]^ Some studies reported that total and subtotal resection had comparable recurrence rates. We believe that the recurrence rate is related primarily to the nature of the tumor and is less likely associated with the resection method. The recurrence incidence of mature teratomas is low, and the probability of recurrence in immature teratomas is high. In the current review, there were no recurrences among the eight mature teratoma cases during the follow-up period regardless of the resection method (total versus subtotal resection). However, the two patients with immature teratomas undergoing total resection and partial resection, respectively, relapsed within a short period of time after surgery.

Histopathology of the resected tissue ultimately determines the definitive diagnosis of teratoma and identifies mature and immature lesions. A typical mature teratoma includes a mixture of well differentiated tissues from the epithelial and mesenchymal elements of the 3 germ layers: endoderm, mesoderm, and ectoderm. The tumor tissue, which is always disorganized, may be composed of less than three germ layers, as derivatives of 1 or 2 layers may overgrow the others.^[15,34]^ Conversely, immature teratomas are composed of poorly differentiated cells and tissues, and some primitive structures may be seen in the lesion. Intramedullary teratomas should be differentiated from other intramedullary lesions such as astrocytomas, ependymomas, hemangioblastomas, schwannomas, neurofibromas, and enterogenous cysts, which have structures resembling a teratoma, which is a source of dilemma for the differentiation of teratomas. Teratomas are subdivided into 3: group I is characterized by the presence of only endodermally derived tissues, group II characterized by endodermally and mesodermally derived tissues, and group III characterized by endodermally, mesodermally, and ectodermally derived tissues.^[11,12]^

There is no consensus on adjuvant therapy for teratomas. For mature teratomas, due to its indolent growth characteristics, the recurrence rate is very low even without postoperative adjuvant radiotherapy or chemotherapy.^[30,32]^ Additionally, whether adjuvant radiotherapy after resection reduces recurrence of immature teratomas remains unclear. Some authors believed that postoperative radiotherapy and chemotherapy may have a role in reducing the recurrence and progression of malignant intramedullary teratoma.^[8]^ In Dr Han's report, they used a dose of 14Gy to 36Gy for local radiotherapy after tumor resection. In addition, they used etoposide phosphate (150 mg, days 1–4) and cisplatin (35 mg, days 1–4) for chemotherapy.^[7]^

One of the most important factors in determining prognosis is the pathological nature of the tumor. In most patients receiving successful resection, postoperative symptoms are relieved to varying degrees, regardless of whether the tumor was benign or malignant.^[12,13]^ In long-term follow-up, the main indicator of prognosis is recurrence, which is closely related to the nature of the tumor, that is, whether it is a mature or immature teratoma.

Although informative, this review was limited by the selection of retrospective case reports. Also, reporting bias and selection bias must be considered because not all cases of ICTs reported in the literature were included, and articles in languages other than English were excluded.

## 5 Conclusion

Our systematic review reveal ICTs as an extremely rare entity that are mainly located in the center or dorsal part of the spinal cord which mainly manifest as quadriplegia and neck pain, accompanied with a skin dimple in the posterior neck that has a tract entering the dura in some cases. MRI is a useful modality that provides diagnostic clues. Surgery from a posterior approach is the primary treatment, and the effect of adjuvant therapy remains uncertain. The prognosis is mainly related to the pathological nature of the tumor and not the method of resection.

## Author contributions

**Data curation:** Tongxiang Li.

**Formal analysis:** Lang Li.

**Investigation:** Fei Xing.

**Methodology:** Min Gong.

**Project administration:** qing guan.

**Software:** Rui Xiao.

**Supervision:** qing guan.

**Writing – original draft:** Lishuai Wang.
